# The role of magnetic resonance-guided focused ultrasound in fertility-sparing treatment of uterine fibroids—current perspectives

**DOI:** 10.3332/ecancer.2020.1034

**Published:** 2020-05-06

**Authors:** Michał Ciebiera, Tomasz Łoziński

**Affiliations:** 1 Second Department of Obstetrics and Gynaecology, The Center of Postgraduate Medical Education, Warsaw, Poland; 2 Department of Obstetrics and Gynaecology, Pro-Familia Hospital, Rzeszów, Poland; a http://orcid.org/0000-0001-5780-5983

**Keywords:** uterine fibroid, leiomyoma, pregnancy, fertility preservation, magnetic resonance-guided focused ultrasound, MRgFUS, magnetic resonance-guided high-intensity focused ultrasound, MR-HIFU

## Abstract

Uterine fibroids (UFs) are the most common benign tumours of the female reproductive system and the most frequent reason for hysterectomy worldwide. UFs are reported in 20%–70% of women of reproductive age depending on a study group. Although most women with UFs are asymptomatic, over 30% of them will present with different symptoms. Abnormal uterine bleeding, pain, pressure and infertility are the most common. Lesions that cause these kinds of symptoms may require medical intervention.

Trends in UF treatment change along with patient awareness and the introduction of new methods and techniques. Selecting an appropriate treatment option should be individualised and adjusted to the patient’s expectations as much as possible. This choice will mostly depend on the patient’s age, UF location, the size and number of lesions, severity of symptoms and, most importantly, the patient’s expectations concerning the preservation of fertility. Observations made for the past few years showed an increasing number of pre- and perimenopausal women who wish to preserve their uterus or decline surgery. In line with current trends and demands in medicine, great importance is attached to the development and upgrade of new minimally invasive or noninvasive procedures in UF therapy.

Magnetic resonance-guided high-intensity focused ultrasound (MR-HIFU) is not associated with severe destruction of the uterine cavity and walls. For this reason, this method may be considered as a kind of hope in fertility-sparing UF therapy and the data about its use in this indication raises future hope. In this review, we summarise the available data on the use of MR-HIFU as a fertility-sparing method in the treatment of UFs. We also indicate how it could evolve in the future.

According to the available data, MR-HIFU is a relatively safe noninvasive method which seems not to deteriorate fertility compared to the pre-treatment status. MR-HIFU may constitute an alternative solution and be chosen in patients who meet the qualification criteria and deny other methods, which also facilitates the use of other treatment options in case the procedure is ineffective. Further randomised studies are necessary to confirm the above information.

## Review

### Uterine fibroids—overview and epidemiology

Uterine fibroids (UFs) are the most common benign tumours of the female reproductive tract and the most frequent reason for hysterectomy worldwide [1]. The lesions are asymptomatic in approximately 50% of cases, while the remaining ones trigger the following manifestations: menorrhagia, dysmenorrhea, anaemia of varying severity, dyspareunia, pelvic pain, psychological disorders and pregnancy complications, including fertility disorders, miscarriage or premature labour [2–4]. UFs are reported in 20%–70% of women of reproductive age depending on a study group [1, 3, 5]. Precise epidemiological data assessing the occurrence of UFs in a population are unavailable, as the majority of studies regarding the frequency present estimations based on clinical diagnosis or imaging work-up [6]. UF occurrence tends to increase with age through the reproductive years and decline in the postmenopausal period [3, 7]. As found by Yu *et al* [8] incidence rates for UF diagnoses are the highest for women between 45 and 49 years of age [8]. Therefore, the pathology should be regarded in a multidimensional spectrum, especially in terms of being a considerable socioeconomic problem of healthcare systems regardless of a country [9]. The occurrence of UFs is estimated at 12.8 per 1,000 person-years for all diagnoses to approximately 2 per 1,000 person-years for cases confirmed after hysterectomy as found by Wise *et al* [3]. Furthermore, as regards the United States, the majority of hospitalisations due to clinically symptomatic UFs involved a surgical procedure.

The causes underlying the development of those tumours have not been fully elucidated and numerous studies are conducted in this matter. According to current knowledge, UFs develop via the conversion of a normal myometrial cell into a mutated UF cell which further divides. Oestrogens and progesterone are hormones considered to play an essential role in promoting the formation and growth of those tumours [10, 11]. The influence of the above-mentioned hormones on a variety of growth factors affects the basic pathophysiological processes by stimulating cell division, growth and storage of extracellular matrix [12]. Genetic factors also play a significant role in UF incidence, as UFs are more common in some populations, e.g., African-American women [5, 13, 14].

### Uterine fibroids—treatment methods

Selecting an appropriate treatment option should be individualised and adjusted to the patient’s expectations as much as possible. It depends on age, location, the size and number of lesions, severity of symptoms and, most importantly, the patient’s expectations concerning the preservation of fertility [1, 15].

Numerous modalities are available—from conservative treatment to less or more invasive surgeries [16]. Conservative treatment methods are more commonly recommended in young women who still have reproductive plans, or in those who refuse operative treatment while wishing to maintain the optimal quality of life [4]. Conservative treatment mostly involves drug administration which reduces symptoms (e.g., bleeding and pain). It may include analgesics, antihaemorrhagic drugs, oral contraceptives, levonorgestrel intrauterine system, gonadotropin releasing hormone (GnRH) analogs or ulipristal acetate (UPA)—a selective progesterone receptor modulator [16]. The effectiveness of the majority of drugs varies for this indication and, to date, most of them have been selected on an empirical basis [17]. GnRH analogs and UPA are currently considered to be the best available drugs. The effectiveness of GnRH analogs in reducing UFs was confirmed a long time ago [18], but numerous patients resigned from this drug due to intensified adverse effects [19]. In case of UPA, the effectiveness is also high and, according to available data, the drug is very effective [16, 20]. However, its administration requires a thorough monitoring of hepatic parameters in order to avoid an increased risk of complications, such as liver failure [21]. The recent problems with UPA constituted the basis of the decision to recall them from market [22].

The surgical treatment of UFs involves tumour removal with endoscopic techniques (laparoscopy and hysteroscopy) or open surgery with the removal of the body of the uterus or even the whole uterus with appendages being the most radical modality in UF treatment, which, fortunately, is performed less and less commonly [16]. The new generation equipment is more often in use in endoscopic facilities. Technological upgrades have made endoscopic methods safer and more effective, especially as regards hysteroscopy. For example, cold loop myomectomy is thought to be the safest method that preserves the anatomic and functional integrity of the myometrium [23]. There are also new hysteroscopic excision and removal technologies, such as morcellators [24] or brand new graspers both for the diagnosis and treatment [25]. The use of systems that use laser light for the enucleation of UF is also a promising option [26].

The trends in UF treatment change along with patient awareness and introducing newer treatment methods. According to Morgan *et al* [27], 386,226 women underwent hysterectomy between the years 2010 and 2013. The study demonstrated that the rate of utilisation decreased by 12.4%, from 39.9 to 35.0 hysterectomies per 10,000 women [27]. It is an emphatic proof of a change in the awareness of physicians who introduce minimally invasive methods more commonly. Fertility-sparing leaves the patient with the option of childbearing plans. Furthermore, observations made for the past few years have shown an increasing number of perimenopausal women who wish to preserve their uterus for reasons other than childbearing. Accurate diagnosis may result in a minor surgery or tailored medical therapy being directed at UFs and may help avoid major surgical treatment [28]. Therefore, it is so important that the offer and selection of treatment should involve surgical methods which are minimally, or even almost noninvasive, such as tumour-supplying uterine artery embolisation (UAE) or using a beam of radio waves during endoscopic procedures, and therapy with magnetic resonance-guided focused ultrasound (MRgFUS/MR-HIFU) [29, 30].

Numerous recommendations are applicable concerning appropriate treatment selection. With regard to the development of new modalities, the recommendations are updated accordingly. To provide an example—in 2015, the Society of Obstetricians and Gynaecologists of Canada (SOGC) proposed an algorithm of UF treatment which included the majority of known forms of treatment. This exemplary algorithm also includes UAE and MRgFUS as essential treatment methods in premenopausal patients who wish to preserve the uterus, but not as options of fertility-sparing [31]. They will be discussed in the subsequent sections.

### Uterine fibroids during preconception and pregnancy

The prevalence of UFs during preconception period and pregnancy is underestimated. The tumours are present in 5% to 10% of infertile patients [32]. Numerous mechanisms of UFs influence fertility. They include implantation disorders, impairment of blood flow in the uterine tissue, endocrine disorders and deceleration or inability to transport gametes [33]. UFs may significantly complicate pregnancy and their incidence during pregnancy increases because of the fact that the number of older mothers is on the rise [34]. The number of pregnancies complicated with UFs depends on ethnicity with the highest percentage occurring in African-American women, as the tumour is the most common in this population [35]. Although data are conflicting and most women with UFs have safe pregnancies, evidence found in the available literature suggests that those tumours are associated with an increased rate of severe obstetric outcomes, including miscarriages, preterm labour and placental abruption [34]. The frequency of complications depends on age. Pregnancy in older patients is characterised by a poorer course and prognosis. Retroplacental location of UFs demonstrates a significant correlation with a higher risk of premature placental abruption, preterm delivery and intrauterine growth restriction. Therefore, the poorest clinical situations seem to occur with UFs located under the placenta of an older patient [34, 36]. Tumour size is proportional to an increased risk of preterm labour and more marked blood loss in the mechanism of uterine atonia [37]. Moreover, a recent study by Karlsen *et al* [38] demonstrated an association between having a UF and time to pregnancy. Due to the lack of data, physicians are unable to inform patients that tumours may modify their size and present clinical symptoms during pregnancy [39]. According to Chill *et al* [40], UFs grow substantially during the first trimester of pregnancy, and this trend changes later with minimal growth towards the end of gestation. The severity and types of symptoms occurring in gestations complicated with UFs have not been subjected to any randomised studies of statistical significance. The issue requires further research. However, it is certain that pregnancy has to be cautiously monitored in the antenatal period to detect any adverse obstetric complications, and so to improve the perinatal outcomes [41].

### Uterine fibroids—treatment before getting pregnant

The hypothesis that prophylactic UF removal increases the probability of conceiving has not been confirmed by relevant research [42]. However, it is not true in case of submucosal or intramural fibroids which exert visible pressure on the line of the endometrium (uterine cavity deformity) as they may affect fertility to some extent. UF treatment should be considered in women in whom other reasons for infertility or miscarriages (infections, uterine defects and disrupted ovulation) were gradually ruled out [43]. In some cases, even if UFs do not trigger symptoms such as typical pain or menorrhagia, further UF management should be discussed with the patient, for example, in order to reduce possible gestational complications [32, 34]. The selection of treatment methods should be presented to the patient in a reliable way showing the advantages and disadvantages [32, 43]. The patient should be familiar with arguments for surgical resection, but she should also know that postoperative adhesions may lead to infertility, miscarriages and the necessity of subsequent surgeries. Surgical resection of UFs in childless women is almost always a choice of ‘the lesser evil’ and the physician’s experience is crucial in selecting the appropriate modality. Patient’s age is a fundamental criterion. In younger asymptomatic women the physician may recommend trying to conceive within a specified timeframe, e.g., a year. If no conception or a miscarriage occurs within the timeframe, surgical treatment may be offered. In submucosal UFs, hysteroscopic myomectomy is nowadays the gold standard [44]. According to the current data, submucosal lesions, especially if they are multiple, may impact implantation and pregnancy outcomes because of uterine cavity abnormalities. Therefore, the Consensus Statement of the Global Congress on Hysteroscopy Scientific Committee declared that when fertility is of highest priority the presence of one or more asymptomatic submucosal UFs ≥15 mm is an indication for the removal [44]. Cold loop systems should be the first-choice approach as they cause less damage. However, other hysteroscopic myomectomy techniques, e.g., morcellation with new generation systems, are also acceptable [24]. Seemingly, it is erroneous to conduct a longlasting (several years) observation of UFs in asymptomatic women who are unable to conceive after excluding other factors affecting fertility (e.g., ovulation, sperm and fallopian tube patency) [32]. It should be remembered that the endometriosis develops more commonly when patient already has UFs. Even with the lack of clinical symptoms, it may lead to impaired fertility as well [45].

The influence of UFs on the course of gestation is determined by UF location, size and location relative to the placenta. When deciding on a surgery in a woman wishing to preserve fertility, it is important to remember that a large surface of the wound after surgical resection may result in adhesion formation, and uterine cavity destruction may lead to the development of intrauterine adhesions [46], which may impede or prevent conceiving and carrying a gestation to term. The patient should be informed about all the possible consequences of a surgery and the resection should be performed by experienced surgeons. The course of the procedure should depend on the skills of the operator, UF size and location. It should not be uncommon to be preceded by the adequate preparation of the patient by the administration of a specific agent [47]. In our viewpoint, the above recommendations need to be complemented with the assessment of the rate and direction of UF growth prior to deciding on treatment implementation. In case of noticeable growth and increased pressure on the uterine cavity shown by subsequent tests, the surgery should not be delayed, even if no significant symptoms are present. The symptoms may develop relatively soon. The lack of an optimal and fully effective method of surgical treatment which allows for complete UF resection with a certainty of preserving fertility translates into more common fear and resignation from surgery and selecting other modalities, e.g., treatment based on a pharmacological [48] or physical model [49]. Noninvasive procedures, such as UAE or MRgFUS/MR-HIFU, which are not associated with severe destruction of the uterine cavity and walls, have been studied by researchers for a long time and are more and more commonly implemented by practitioners.

UAE was commonly described along with MRgFUS/MR-HIFU as accessory methods of UF treatment. Therefore, this opinion also includes UAE-related information. Several years ago those methods were used for similar indications, but currently differences are noted in available data. UAE was first used in UF treatment in France in 1995 [50]. It is one of recommended methods in the treatment of symptomatic UFs by various gynaecological societies, such as the Royal College of Obstetricians and Gynaecologists [51] or previously mentioned SOGC [31]. MRgFUS/MR-HIFU is becoming more common in patients of reproductive age, while UAE is introduced in women who do not wish to conceive. UAE procedure involves the obliteration of tumour vessels by an interventional radiologist which leads to acute tumour necrosis [29, 50]. It frequently leads to severe pain or infections [52]. UAE may disrupt uterine cavity vascularity, indirectly and endometrial receptivity. It also impairs ovarian vascularity [53]. As previously mentioned, no relevant studies are available to tackle the issue of fertility following embolisation. Therefore, experts recommend uttermost cautiousness and reliable information provision to those patients who plan to conceive [54].

### Uterine fibroids—treatment with focused ultrasound as a fertility-sparing method

In our viewpoint, MRgFUS/MR-HIFU technique is a still fresh player in the group of noninvasive methods of fertility-sparing in UF treatment. The energy of focused ultrasound beam under the control of modern magnetic resonance imaging (MRI) devices (also ultrasound-guided) makes the technique highly precise. The planned clinical effect is to some extent similar to that of UAE, as the method also results in tumour necrosis. However, the precision of the procedure is considerably higher, as it targets specific tissue and not a vessel which may include numerous collaterals [55, 56]. How is UF tissue destroyed? High-frequency ultrasound waves cause an increase in the temperature, secondary protein denaturation and insufficient blood supply [57, 58].

MRgFUS differs from MR-HIFU, which is a more recent generation of the procedure. The former technique involves heating tumour surface, while in MR-HIFU the whole procedure involves adequate volumetric heating of UFs with real-time feedback which makes it even more precise. Heating every part of the tissue that has been targeted and ablated volumetrically makes it more efficient and quicker [49, 59]. In MR-HIFU, the tumour is divided into small sections which are subsequently subjected to ultrasound beam activity. However, it makes the procedure relatively time-consuming. Seemingly, the energy of ultrasound beam used in HIFU may be insufficient to cause a permanent destruction of the endometrium. It is corroborated by findings of a study which described gestations following submucosal UF ablation associated with increased temperatures in the uterine cavity and by a case report which we presented describing an early pregnancy in the uterine cavity of a patient who underwent MR-HIFU [60]. Moreover, highly precise targeting of ultrasound waves on tumour tissue reduces the risk of destroying adjacent tissues. Therefore, it may be expected that the method may be used in selected women with UFs who have fertility problems. Interestingly, Keserci and Duc [61] reported that the level of anti-Mullerian hormone tested 6 months after a HIFU procedure did not decrease and was not different from the level in the control group. It indicated that ovarian vessels had not been destroyed during the procedure. The authors concluded that the achievement of non-perfused volume ratio of at least 90% during MR-HIFU treatment of UFs based on the prediction model appears clinically possible without compromising the safety. Obviously, not all the patients who underwent MR-HIFU procedures may conceive. It seems that the inability to conceive after the procedure may be associated with the fact that the tumour remaining in the uterine wall is smaller, but it may still disrupt the contractility of the myometrium and endometrial vascularity [62]. Regrettably, despite a high effectiveness of the procedure, the percentage of patients disqualified remains high because of unsatisfactory conditions. Factors which contribute to the positive result of qualification include: tumour location, its location relative to the adjacent structures (especially to the intestine), adipose tissue thickness and the MRI image according to criteria by Funaki *et al* [63]. Currently, the number of qualification criteria reduces the treatment qualification rates to only 25%–30% of UFs.

An increasing number of authors (including our centre) describe gestations which were carried to term despite MR-HIFU treatment [49, 64]. Rabinovici *et al* [65] described 51 gestations in UF patients treated with thermal ablation. The numbers are constantly increasing and the majority of data comes from the East. Zou *et al* [66] has recently reported on a large group of patients treated with ultrasound-guided HIFU in the years 2011–2016, with 80 gestations which were achieved. Interestingly, the Chinese authors recently found out that vaginal delivery may be preferable following a HIFU procedure. It is due to the fact that, unlike with surgery, adhesions are not formed, and there is no risk of uterine wall rupture during gestation and delivery [64]. Our experience is also considerable in this matter. In 2019, we published a study describing patients who conceived after MR-HIFU therapy. General improvement and the alleviation of symptoms of the disease were observed after the procedure in the majority of the patients. Most importantly, our experience showed that MR-HIFU did not increase the rate of spontaneous abortions or pregnancy complications [49]. Additionally, we published a case report in which a patient with a very early pregnancy underwent MR-HIFU treatment and developed no complications associated with the therapy [60].

A detailed classification of MR-HIFU method is problematic and results from the lack of randomised studies. The lack of studies and randomisation are the reasons why societies of gynaecologists and radiologists do not recommend the method to patients who wish to conceive [67]. Such studies are immensely difficult, if not impossible to conduct, due to various patient expectations and various healthcare systems. Unsatisfactory cooperation between radiologists and gynaecologists also remains problematic. Moreover, the optimal construction of referral system is of high importance [68]. Professional literature is dominated by comparative studies on surgical treatment. Therefore, the comparison of MRgFUS/MR-HIFU groups with surgical ones does not offer appropriate statistical significance. It is obviously due to the enormous disproportion between the number of gynaecological centres which perform UF surgeries and a low number of interventional radiology centres which are equipped with devices used in methods presented above. Thermal ablation devices are very expensive and the duration of the procedure additionally limits the possibility of using MRI for the benefit of other patients at the same time. The procedure is performed by a specially trained interventional radiologist under the supervision and in close cooperation with a gynaecologist, which constitutes another organisational challenge for the hospital and significantly increases the costs.

UF removal may be implemented in almost all kinds of UFs, both solitary and multiple ones, with the scope and type of operative procedure being determined solely by the experience and judgment of the operator. In case of other methods, including MR-HIFU, a marked limitation is encountered due to the anatomy, size, location and type of UFs. Furthermore, the cost of the procedure should also be considered, as its financing is of key importance. Surgical methods are approved and refunded by insurance companies and institutions [69], while MRgFUS/MR-HIFU is currently refunded only in Israel and Canada. To conclude, it may be stated that current evidence supports that myomectomy may still be a better choice for women who desire to have a child. However, treatment choice will also be dictated by the location, size, and the number of UFs, previous surgery or operative risk, and in selected cases MR-HIFU may be the best approach.

### Focused ultrasound as a fertility-sparing method in oncology?

The diagnosis of a gynaecological cancer has a strong negative impact on female sexuality. Psychological functioning is affected by this diagnosis and implemented therapies may compromise the reproductive function [70]. Over the years, MRgFUS/MR-HIFU has also been found effective not only in benign lesions, but also in noninvasive ablative treatment of malignant tumours (71). As this method is capable of providing noninvasive treatment to a small target volume without causing damage to the adjacent tissues, some trials about its use as alternative oncological treatment are also available. MRgFUS/MR-HIFU offers a great quality of targeting. The temperature feedback allows the direct delivery of a defined dose of thermal ablation to the malignant tissue. Nowadays, new transducers are developed to provide the treatment in several malignancies, including prostate or breast cancers [72]. Other indications of MRgFUS/MR-HIFU use might include the palliation of pain due to tumour occurrence or metastasis by producing tissue denervation, tumour mass reduction and neuromodulation [73]. In a study by Catane *et al* [74], most patients received adequate treatment and had prolonged improvement in the pain score. The authors concluded that this method may then be an alternative to bone metastasis pain relief in oncological patients.

Currently available data and database search showed that not much data may be retrieved about the use of HIFU in gynaecological cancers, except the breast. As for fertility-sparing methods, we see some chance of the use of this method in early-stage endometrial cancer. Some data are available from animal (rabbit) studies in this indication. According to Guan *et al* [75], HIFU could be a minimally invasive method that may destroy all endometrial cancer cells and their blood vessels. Therefore, it may be an alternative approach of targeted therapy in endometrial cancer. Further studies on humans might very interesting and we are waiting for further trials in this field.

The main problem associated with MRgFUS/MR-HIFU in oncology is the question of the radicality. It is still believed that complete pathological ablation is not always obtained in all patients with malignant lesions. As concluded by experts, the techniques and equipment should be further developed and upgraded in order to achieve the best level of treatment and only large prospectively conducted clinical trials might validate the efficacy of HIFU in comparison with surgery [71]. According to ClinicalTrials.gov by U.S. National Library of Medicine, the use of MR-HIFU in gynaecological oncology was subjected to trials. However, they do not determine the use of this method as primary treatment, but its use in recurrent pelvic malignancy with an acceptable safety profile in patients who cannot receive conventional treatment [76].

## Conclusions

Numerous modalities are available for women with UFs who wish to conceive. The selection of a method needs to be preceded by a thorough analysis of the case, patient’s age, tumour location and related symptoms. MR-HIFU (also ultrasound-guided HIFU) is a relatively safe noninvasive method which seems not to deteriorate fertility compared to pre-treatment status; however, there is still a lack of good-quality data. In our viewpoint, MR-HIFU may constitute an alternative solution in patients who deny surgical treatment and be chosen in patients who meet the qualification criteria which also facilitates the use of other treatment options in case the procedure is ineffective. Further randomised studies are necessary to confirm the above information. If such studies are not conducted it seems justified to inform patients about the possibility of undergoing such a therapy prior to the implementation of other treatment forms. Additionally, the patients should be informed about the positive results obtained in early phase studies.

## List of abbreviations

HIFU: High-intensity guided ultrasound
GnRH: Gonadotropin releasing hormone
MRgFUS: Magnetic resonance-guided focused ultrasound
MR-HIFU: Magnetic resonance-guided high-intensity focused ultrasound
MRI: Magnetic resonance imaging
SOGC: Society of Obstetricians and Gynecologists of Canada
UAE: Uterine artery embolisation
UF: Uterine fibroid

## Conflicts of interest

All authors declare that they have no conflicts of interest.

## Funding

The authors did not receive any specific funding regarding this manuscript.
